# A mathematical model for the simulation of the formation and the subsequent regression of hypertrophic scar tissue after dermal wounding

**DOI:** 10.1007/s10237-016-0799-9

**Published:** 2016-05-26

**Authors:** Daniël C. Koppenol, Fred J. Vermolen, Frank B. Niessen, Paul P. M. van Zuijlen, Kees Vuik

**Affiliations:** 1 Delft Institute of Applied Mathematics, Delft University of Technology, Delft, The Netherlands; 2 Department of Plastic, Reconstructive and Hand Surgery, MOVE Research Institute, VU University Medical Centre, Amsterdam, The Netherlands; 3 Burn Centre, Red Cross Hospital, Beverwijk, The Netherlands; 4 Department of Plastic, Reconstructive and Hand Surgery, Red Cross Hospital, Beverwijk, The Netherlands

**Keywords:** Dermal wound healing, Hypertrophic scar tissue, Fibroblasts, Compressible neo-Hookean solid, Modeling, Biomechanics, Moving boundary, Moving-grid finite-element method, Flux-corrected transport (FCT) limiter, 35L65, 35M10, 65C20, 68U20, 74L15, 92C10, 92C17

## Abstract

A continuum hypothesis-based model is presented for the simulation of the formation and the subsequent regression of hypertrophic scar tissue after dermal wounding. Solely the dermal layer of the skin is modeled explicitly and it is modeled as a heterogeneous, isotropic and compressible neo-Hookean solid. With respect to the constituents of the dermal layer, the following components are selected as primary model components: fibroblasts, myofibroblasts, a generic signaling molecule and collagen molecules. A good match with respect to the evolution of the thickness of the dermal layer of scars between the outcomes of simulations and clinical measurements on hypertrophic scars at different time points after injury in human subjects is demonstrated. Interestingly, the comparison between the outcomes of the simulations and the clinical measurements demonstrates that a relatively high apoptosis rate of myofibroblasts results in scar tissue that behaves more like normal scar tissue with respect to the evolution of the thickness of the tissue over time, while a relatively low apoptosis rate results in scar tissue that behaves like hypertrophic scar tissue with respect to the evolution of the thickness of the tissue over time. Our ultimate goal is to construct models with which the properties of newly generated tissues that form during wound healing can be predicted with a high degree of certainty. The development of the presented model is considered by us as a step toward their construction.

## Introduction

The skin is an important, multifunctional organ; it plays a crucial role in maintaining homeostasis and acts as a protective physical barrier to potentially damaging environmental factors such as pathogens, oxidative stresses and applied mechanical forces (O’Toole and Mellerio 2010). This implies that dermal wounds may cause a variety of potentially lethal pathological conditions, and therefore, the speedy and effective restoration of the integrity of affected skin is crucial. In order to accomplish this, humans have evolved sophisticated processes for the healing of wounds. In the case of deep dermal wounds, the final outcome of the execution of these processes is usually a thin, flat scar when the restoration of the wounded area proceeds without complications (Hawkins and Finnerty 2012; Verhaegen et al. 2009).

Many times, however, the restoration of these deep dermal wounds gets perturbed during the initial period post-wounding, and this might cause the growing, newly generated tissue to evolve into hypertrophic scar tissue (Hawkins and Finnerty 2012; Niessen et al. 1999). There are many factors that influence the properties of the evolving hypertrophic scar tissue, but in general the growing tissue becomes firm and thick and has a dark red appearance. After the initial expansion phase, the hypertrophy of the tissue usually regresses gradually over an extended period of time, and ultimately, the outcome of the perturbed restoration process is ordinarily a relatively flat and inflexible scar (Hawkins and Finnerty 2012).

The causal pathways that lead to hypertrophic scar tissue formation are unknown at present (Van der Veer et al. 2009). As a consequence, it is very difficult in general to predict the properties of the newly generated tissues that form during the healing of deep dermal wounds. Given that a high-quality prediction most likely would improve both the prevention of hypertrophic scar tissue formation and the treatment of its symptoms and exterior features, it is very unsatisfactory that such a prediction cannot be made. Mathematical models might aid in making these high-quality predictions possible, and with the development of the mathematical model that is presented in this study, we think we made a further step toward the construction of such predictive models.

Before presenting the model, we present in Sect. 2 a general overview of the main processes that bring about the formation of normal scar tissue during the healing of deep dermal wounds that cover a large surface area. This overview serves as the biomedical basis for the development of the model. Subsequently, we develop the model in Sect. 3. In Sect. 4, we give a broad overview of the numerical algorithm that has been used for obtaining simulation results. These results are presented in Sect. 5. Finally, the model and the simulation results are discussed in Sect. 6.

## Dermal wound healing: an overview

As has been mentioned in the introduction, the final outcome of the restoration of a deep dermal wound is usually a thin, flat scar when the restoration of the affected skin proceeds without complications. The processes that bring about the formation of this type of scar tissue are often divided up in four sequential, partially overlapping phases: hemostasis, inflammation, proliferation and maturation/remodeling (Enoch and Leaper 2007; O’Toole and Mellerio 2010).

### Hemostatic phase

Wound healing starts with hemostasis. Hemostasis is the process that causes a bleeding to stop. It consists of three subprocesses: vasoconstriction of nearby blood vessels, adhesion and aggregation of platelets, and deposition of a provisional fibrin-based extracellular matrix (ECM) (Baum and Arpey 2005). While bleeding stops, activated platelets start releasing the contents of different types of granule into the extracellular space. These granules contain several chemical substances such as proteins that contribute to the formation of the provisional matrix (Hynes 1986) and different types of signaling molecule (i.e., growth factors and cytokines) that influence the behavior of for instance leukocytes, fibroblasts and endothelial cells by stimulating among other things chemotaxis, cell differentiation and cell division (Barrientos et al. 2008; Werner and Grose 2003).

### Inflammatory phase

The initial vasoconstriction of the nearby blood vessels is reversed quickly after injury and is succeeded by vasodilatation and increased permeability of the walls of these vessels (Baum and Arpey 2005; Monaco and Lawrence 2003). These changes are brought about by the complex and highly regulated interplay between the nervous system and various signaling molecules. Effectively, this results in the leakage of plasma from the intravascular space to the extravascular space and the active wound infiltration of various types of leukocyte such as polymorphonuclear leukocytes (PMNs), monocytes, macrophages (activated and transformed monocytes) and T lymphocytes. The active infiltration is mediated through the presence of a variety of chemoattractants in the wounded area (which are in part present here due to the release of the contents of the aforementioned granules) and marks the start of the inflammatory phase (Lawrence 1998; Singer and Clark 1999).

Early in the wound healing cascade, macrophages and neutrophils are the dominant types of leukocyte in the injured area (with neutrophils arriving first) (Delavary et al. 2011; Eming et al. 2007). After activation, these cells start cleaning the wounded area by removing bacteria and debris through phagocytosis and the release of different types of metalloproteinase (MMP). Besides cleaning the wounded area, these cells also secrete various signaling molecules. These signaling molecules are very important for successful completion of the wound healing cascade because of the fact that they perpetuate the inflammatory response, stimulate angiogenesis, and affect both fibroblasts and keratinocytes in various processes such as protein production, cell division and cell migration (Li et al. 2007).

T lymphocytes become the dominant type of leukocyte in the wounded area during the later stages of the wound healing cascade (Eming et al. 2007). Results from previous studies have demonstrated that the removal of all T lymphocyte subtypes from the injured area results in impaired healing (Barbul et al. 1989; Efron et al. 1990). Hence, this type of cell is apparently very important for a successful completion of the wound healing cascade. T lymphocytes are the main effectors of the cell-mediated immune response and are major sources of signaling molecules that regulate important processes such as the cell division and the cell differentiation of various cell types present in wounded area (Monaco and Lawrence 2003).

### Proliferative phase

Soon after the initiation of the inflammatory phase, the proliferative phase of the wound healing cascade commences (Enoch and Leaper 2007). The subprocesses that take place during the proliferative phase are reepithelialization, angiogenesis, wound contraction and fibroplasia (Monaco and Lawrence 2003; Li et al. 2007; Singer and Clark 1999).

Reepithelialization encompasses the subprocesses that bring about the restoration of an intact epidermis, and angiogenesis comprises a sequence of subprocesses through which new capillaries in the wounded area are formed from preexisting blood vessels. Due to the restoration of the epidermis, a crucial part of the protective physical barrier is restored. The renewed presence of blood vessels in the injured area is very important since it improves the delivery of nutrients and oxygen to the reconstituting dermal tissue, and contributes to the enhancement of the influx of leukocytes and other cell types by providing both more and more proximate locations for these cells to infiltrate the wounded area.

Wound contraction is the process that causes the circumferential inward movement of surrounding uninjured skin tissue toward the injured area (O’Toole and Mellerio 2010). The amount of contraction is dependent on the size, shape, depth and anatomical location of the wound (Baum and Arpey 2005; Li et al. 2007; Monaco and Lawrence 2003). Due to the contraction of the wounded area, the exposed surface area of the wound is decreased relatively fast without the production of new wound-covering tissue. This is advantageous in general given that the production of mature scar tissue of sufficient quality takes longer and fast closure of the wounded area is necessary so that the influx of, for instance, bacteria is minimized as much as possible. Minimizing this influx aids subsequently in accelerating the wound healing cascade.

Two theories exist that can both explain the compaction of the wounded area. On the one hand, there is the myofibroblast theory (Gabbiani et al. 1971), while on the other hand, there is the fibroblast theory (Harris et al. 1981). According to the myofibroblast theory, myofibroblasts are primarily responsible for the contraction of the wound. Myofibroblasts are modulated fibroblasts that are among other things characterized by the presence of actin microfilament bundles in their cytoskeleton, similar to those observed in smooth muscle cells (Tomasek et al. 2002). Over the years, numerous studies have demonstrated the presence of myofibroblasts in the wounded area during the execution of the wound healing cascade (Hinz 2007). Due to the actin microfilament bundles, the myofibroblasts can pull at the ECM with relatively much force. Given their position within the wounded area, this can cause effectively the reduction of the surface of the wounded area.

According to the fibroblast theory, the contraction of the wound is accomplished through many individual fibroblasts that exert traction forces on the collagen fibers as they migrate into the wounded area. At this moment, it is impossible to determine *in vivo* which theory is correct. It might very well be the case that both theories are partly correct in that both fibroblasts and myofibroblasts influence wound contraction in different ways and at different times during the wound healing cascade.

Fibroplasia encompasses the subprocesses that cause the restoration of the presence of fibroblasts and the restoration of a collagen-rich ECM in the injured area. Traditionally, it was thought that the repopulation of the wounded area by fibroblasts is realized through the active migration of nearby fibroblasts into the evolving ECM and the cell division of present fibroblasts in this matrix (Lawrence 1998). However, experimental evidence gathered over the last 20 years suggests that this repopulating population of fibroblasts probably accrues from a variety of sources. Besides containing fibroblasts that originate from nearby, uninjured tissue, the population might consist of differentiated cells of the epidermis and the inner lining of blood vessels, differentiated bone marrow- and tissue-derived mesenchymal stem cells, differentiated pericytes and differentiated fibrocytes (Abe et al. 2001; Gharzi et al. 2003; Kalluri and Neilson 2003; Kwan et al. 2012; Mori et al. 2005; Quan et al. 2004).

Taken together, the heterogeneous population of fibroblasts and myofibroblasts is responsible for the adjustment of other wound healing processes through the release of signaling molecules and is the main producer of the constituents of the newly formed collagen-rich ECM (Barrientos et al. 2008; Baum and Arpey 2005; Werner and Grose 2003). The fibrils of this collagen-rich ECM serve two purposes; they provide increased strength to the wounded area and they facilitate, in conjunction with glycoproteins, the migration of cells, such as endothelial cells by providing scaffolding and contact guidance (Monaco and Lawrence 2003).

### Remodeling phase

With the onset of the proliferative phase, the remodeling of the evolving ECM also commences (Enoch and Leaper 2007). However, contrary to the proliferative phase, which is relatively short under normal circumstances, the remodeling of the ECM takes place over a much longer period of time. The subprocesses that underlie the remodeling process are active mostly during the first year post-wounding, but they remain active thereafter (Li et al. 2007). During remodeling, the nature of the ECM changes as a consequence of alterations in the balances between the production and the breakdown of various constituents of the ECM and as a consequence of adjustments in the way that these constituents are aligned and interconnected (Monaco and Lawrence 2003).

For instance, the total amount of collagen and the relative levels of fibronectin, proteoglycans and type III collagen decrease over time, while the relative levels of type I collagen increase over time. Meanwhile, the initial disorganized mesh of newly formed, delicate collagen fibers is replaced by a mesh consisting of thicker, extensively cross-linked collagen fibers that are oriented more parallel to the skin surface. Additionally, the cell densities of various cell types, such as endothelial cells and (myo-) fibroblasts, decrease. Effectively, these alterations result in a relative acellular and avascular, flat and thin scar of gradually increasing strength (Hawkins and Finnerty 2012).

## Development of the mathematical model

In order to simulate the formation and the subsequent regression of hypertrophic scar tissue, we incorporate into the model some of the processes that take place during the proliferative and the remodeling phase of the wound healing cascade. Solely the dermal layer of the skin is modeled explicitly, and this layer is modeled as a continuum. The adjacent subcutaneous layer is incorporated implicitly into the model through a mechanical interaction between this layer and the dermal layer at their interface.

Due to the fact that biological materials, such as skin tissue, are generally nonlinear, anisotropic, viscoelastic and inhomogeneous materials, these tissues exhibit very complex constitutive behaviors (Fung 1993). Hence, the constitutive stress–strain relations for tissues such as granulation tissue, dermal tissue and scar tissue, if these were available, would be very complicated (Bischoff et al. 2004). In order to keep the model as simple as possible while allowing for finite strains, we decided to model the dermal layer as a heterogeneous, isotropic and compressible neo-Hookean solid (Treloar 1948). The displacement of the dermal layer ($\mathbf {u}$) is selected as the primary model variable.

Additionally, we select the following four components of the (healing) dermal layer as primary model components: a generic fibroblast population (*N*), a generic myofibroblast population (*M*), a generic signaling molecule (*c*) and the collagen molecules ($\rho$).

Mathematical modeling of the processes involved in the wound healing cascade has been an active area of research for approximately the last 25 years. During this period of time, the number of mathematical models has increased dramatically along with the general level of complexity. Over the years, several surveys of models have appeared, such as those compiled in Sherratt and Dallon (2002), Geris *et al.* (2010), Buganza Tepole and Kuhl (2013) and Valero *et al.* (2015). These surveys indicate that the majority of the models can be placed into one of the three categories: continuum hypothesis-based models, discrete cell-based models and hybrid models. We use the general continuum hypothesis-based modeling framework of Tranquillo and Murray (1992) as the mathematical basis for the model of this study. This framework consists of the following general set of conservation equations:

1a $$\begin{aligned}&\frac{\partial z_{i}}{\partial t} + \nabla \cdot (z_{i}\mathbf {v}) = -\nabla \cdot \mathbf {J}_{i} + R_{i}, \end{aligned}$$

1b $$\begin{aligned}&-\nabla \cdot \mathbf {\sigma } = \mathbf {f}. \end{aligned}$$

Equation (1a) is the conservation equation for the cell density/concentration of constituent *i* of the dermal layer, and Eq. (1b) is the reduced conservation equation for the linear momentum of the dermal layer. Like others, we assume that the inertial forces that work on the dermal layer are negligible (Murphy et al. 2012; Olsen et al. 1995a; Tranquillo and Murray 1992; Vermolen and Javierre 2012). As a consequence, the conservation equation for the linear momentum of the dermal layer reduces to the above force balance equation. Within the above equations, $z_{i}$ represents the cell density/concentration of constituent *i*, $\mathbf {v}$ represents the displacement velocity of the dermal layer, $\mathbf {J}_{i}$ represents the flux associated with constituent *i* per unit area due to random dispersal, chemotaxis and other possible fluxes, $R_{i}$ represents the chemical kinetics associated with constituent *i*, $\mathbf {\sigma }$ represents the Cauchy stress tensor associated with the dermal layer, and $\mathbf {f}$ represents the total body force working on the dermal layer. Given the chosen primary model variables, we have $i \in \{N,M,c,\rho \}$. In order to simplify notation, we replace $z_{i}$ by *i* in the remainder of this study. Hence, $z_{N}$ becomes *N*, $z_{M}$ becomes *M* and so on.

### The force balance

Given that we model the dermal layer as a heterogeneous, isotropic and compressible neo-Hookean solid, we take the following constitutive stress–strain relation:

2 $$\begin{aligned} J\mathbf {\sigma }&= \left( 2D_{1}J(J - 1)\right) \mathbf {I} + 2C_{1}J^{-\frac{2}{3}}\left( \mathbf {B} - \frac{1}{3}\text {tr}\left( \mathbf {B}\right) \mathbf {I}\right) , \end{aligned}$$

3 $$\begin{aligned} \mathbf {B}&= \left( -2\mathbf {e} + \mathbf {I}\right) ^{-1}, \end{aligned}$$

4 $$\begin{aligned} \mathbf {e}&= \frac{1}{2}\left( \nabla \mathbf {u} + \left( \nabla \mathbf {u}\right) ^{\text {T}} - \left( \nabla \mathbf {u}\right) ^{\text {T}}\nabla \mathbf {u}\right) , \end{aligned}$$

5 $$\begin{aligned} 2C_{1}&= \frac{E(\rho )}{2(1 + \nu )}, \end{aligned}$$

6 $$\begin{aligned} 2D_{1}&= \frac{E(\rho )}{3(1 - 2\nu )}, \end{aligned}$$

7 $$\begin{aligned} E(\rho )&= E^{I}\sqrt{\rho }, \end{aligned}$$

where $J = \sqrt{\text {det}(\mathbf {B})}$, $\mathbf {B}$ is the left Cauchy–Green deformation tensor, $\mathbf {e}$ is the Eulerian finite strain tensor, $E(\rho )$ is the collagen molecule concentration-dependent Young’s modulus (Ramtani 2004; Ramtani et al. 2002), $\nu$ is Poisson’s ratio, and $\mathbf {I}$ is the second-order identity tensor.

Furthermore, we incorporate into the model that the cells from the heterogeneous myofibroblast population are pulling uniformly on their surroundings, both when they are immobile and when they are moving around through the dermal layer. In order to keep the model as simple as possible, we model the pulling force as an isotropic stress that is proportional to the product of the cell density of the myofibroblast population and a simple function of the concentration of the collagen molecules (Olsen et al. 1995b). No other forces are incorporated into the model. Taken together, we obtain

8 $$\begin{aligned}&\mathbf {f} = \nabla \cdot \mathbf {\psi }, \end{aligned}$$

9 $$\begin{aligned}&\mathbf {\psi } = \xi M\left( \frac{\rho }{R^2 + \rho ^{2}}\right) \mathbf {I}, \end{aligned}$$

where $\mathbf {\psi }$ is the total generated stress by the myofibroblast population, $\xi$ is the generated stress per unit cell density and unit collagen molecule concentration, and *R* is a constant.

### The fibroblast population

We incorporate into the model the random movement of fibroblasts through the dermal layer and the directed movement of fibroblasts up the gradient of signaling molecule *c*, if present. The former process is modeled by cell density-dependent Fickian diffusion, and the latter process is modeled by using a very simple model for chemotaxis (Hillen and Painter 2009). Taken together, we obtain

10 $$\begin{aligned} \mathbf {J}_{N} = -D_{F}F\nabla N + \chi _{F}N\nabla c, \end{aligned}$$

with

11 $$\begin{aligned} F = N + M. \end{aligned}$$

$D_{F}$ is the cell density-dependent (myo-) fibroblast random motility coefficient, and $\chi _{F}$ is a chemotactic parameter that depends on both the binding rate and the unbinding rate of the signaling molecule *c* with its receptors and the concentration of these receptors on the cell surface of the (myo-) fibroblasts. A good example of a family of molecules that acts as a strong attracting stimulus for fibroblasts during dermal wound healing is the family of platelet-derived growth factors (PDGF) (Barrientos et al. 2008).

Furthermore, we incorporate into the model the cell division of fibroblasts by using an adjusted logistic growth model and the cell differentiation of fibroblasts into myofibroblasts under the influence of the signaling molecule *c*. The rate of cell division is enhanced in the presence of the signaling molecule. Examples of signaling molecules that can stimulate both the up-regulation of the cell division rate of fibroblasts and the cell differentiation rate of fibroblasts into myofibroblasts are certain members of the family of transforming growth factors $\beta$ (TGF-$\beta$) (Werner and Grose 2003). Finally, we incorporate into the model the removal of fibroblasts from the dermal layer by means of apoptosis. Taken together, we obtain

12 $$\begin{aligned} R_{N} = r_{F}\left( 1 + \frac{r_{F}^{\max }c}{a_{c}^{I} + c}\right) (1 - \kappa _{F}F)N^{1 + p} - k_{F}cN - \delta _{N}N, \end{aligned}$$

where $r_{F}$ is the cell division rate, $r_{F}^{\max }$ is the maximum factor with which the cell division rate can be enhanced due to the presence of the signaling molecule, $a_{c}^{I}$ is the concentration of the signaling molecule that causes the half-maximum enhancement of the cell division rate, $\kappa _{F}F$ represents the reduction in the cell division rate due to crowding, *p* is a constant whose value follows from the equilibrium cell density of the fibroblasts in the unwounded dermis (See Appendix 1), $k_{F}$ is the signaling molecule-dependent cell differentiation rate of fibroblasts into myofibroblasts, and $\delta _{N}$ is the apoptosis rate of fibroblasts.

### The myofibroblast population

We incorporate into the model the random movement of myofibroblasts through the dermal layer and the directed movement of myofibroblasts up the gradient of signaling molecule *c*, if present. We model these processes in the same way as we model these processes for fibroblasts. Hence we get

13 $$\begin{aligned} \mathbf {J}_{M} = -D_{F}F\nabla M + \chi _{F}M\nabla c. \end{aligned}$$

Furthermore, we incorporate into the model the cell division of myofibroblasts by using nearly the same adjusted logistic growth model as used for the fibroblast population. The only difference is that we assume that myofibroblasts solely divide when the generic signaling molecule is present. Finally, we incorporate into the model the removal of myofibroblasts from the dermal layer by means of apoptosis. Taken together, we obtain

14 $$\begin{aligned} R_{M}= & {} r_{F}\left( \frac{\left( 1 + r_{F}^{\max }\right) c}{a_{c}^{I} + c}\right) (1 - \kappa _{F}F)M^{1 + p} \nonumber \\&+\, k_{F}cN - \delta _{M}M, \end{aligned}$$

where $\delta _{M}$ is the apoptosis rate of myofibroblasts.

### The generic signaling molecule

We assume that both fibroblasts and myofibroblasts release and consume the signaling molecules. The functional forms for these processes are based on the interactions between cell surface receptor molecules and the signaling molecules. The derivation of these functional forms can be found in the article by Olsen and colleagues (1995b). Additionally, we incorporate into the model that the signaling molecules are removed from the dermal layer through proteolytic breakdown. Finally, we assume that the signaling molecules diffuse through the dermal layer according to linear Fickian diffusion. Taken together, this results in

15 $$\begin{aligned} \mathbf {J}_{c}&= -D_{c}\nabla c, \end{aligned}$$

16 $$\begin{aligned} R_{c}&= \frac{k_{c}\left( N + \eta M\right) c}{a_{c}^{II} + c} - \delta _{c}g(F,c,\rho )c, \end{aligned}$$

where $D_{c}$ is the Fickian diffusion coefficient of the generic signaling molecule, $k_{c}$ is the maximum net secretion rate of the signaling molecule, $\eta$ is the ratio of myofibroblasts to fibroblasts in the maximum net secretion rate of the signaling molecule and the collagen molecules (See the next subsection), $a_{c}^{II}$ is the concentration of the signaling molecule that causes the half-maximum net secretion rate of the signaling molecule, and $\delta _{c}$ is the breakdown rate of the signaling molecules.

The last term of $R_{c}$ requires some more explanation. We incorporate into the model the proteolytic cleavage of the signaling molecule by a generic MMP (Mast and Schultz 1996; Van Lint and Libert 2007). It is known that MMPs are involved in the breakdown of collagen-rich fibrils during the remodeling of the ECM and the maintenance of the ECM (Chakraborti et al. 2003; Lindner et al. 2012; Nagase et al. 2006). Furthermore, it is known that (myo-) fibroblasts are important producers of MMPs (Lindner et al. 2012) and that the production of MMPs is reduced in the presence of signaling molecules like TGF-$\beta$ (Overall et al. 1991). Therefore, we assume that the concentration of the generic MMP that is responsible for the proteolytic cleavage of the signaling molecule, is a function of the concentration of the collagen molecules, the concentration of the signaling molecule and the cell density of the (myo-) fibroblast population. In this study, we take the following relationship:

17 $$\begin{aligned} g(F,c,\rho ) = \frac{F\rho }{1 + a_{c}^{III}c}, \end{aligned}$$

where $1/(1 + a_{c}^{III}c)$ is the inhibition of the synthesis of the generic MMP due to the presence of the signaling molecule.

### The collagen molecules

We assume that secreted collagen molecules are attached to the ECM instantly, so no active transportation of the collagen molecules takes place in the model. Furthermore, we incorporate into the model that the collagen molecules are produced by both fibroblasts and myofibroblasts. In addition, we include that the secretion rate is enhanced in the presence of the signaling molecule. A good example of a signaling molecule that can bring about this behavior in both fibroblasts and myofibroblasts is TGF-$\beta$ (Werner and Grose 2003). Finally, we incorporate into the model the proteolytic breakdown of the collagen molecules analogously to the removal of the signaling molecules. Taken together, we obtain

18 $$\begin{aligned} \mathbf {J}_{\rho }= & {} \mathbf {0}, \end{aligned}$$

19 $$\begin{aligned} R_{\rho }= & {} k_{\rho }\left( 1 + \left( \frac{k_{\rho }^{\max }c}{a_{c}^{IV} + c}\right) \right) \left( N + \eta M\right) \nonumber \\&\quad -\,\delta _{\rho }g(F,c,\rho )\rho , \end{aligned}$$

where $k_{\rho }$ is the collagen molecule secretion rate, $k_{\rho }^{\max }$ is the maximum factor with which the secretion rate can be enhanced due to the presence of the signaling molecule, $a_{c_{N}}^{IV}$ is the concentration of the signaling molecule that causes the half-maximum enhancement of the secretion rate, and $\delta _{\rho }$ is the degradation rate of the collagen molecules.

**Fig. 1:**
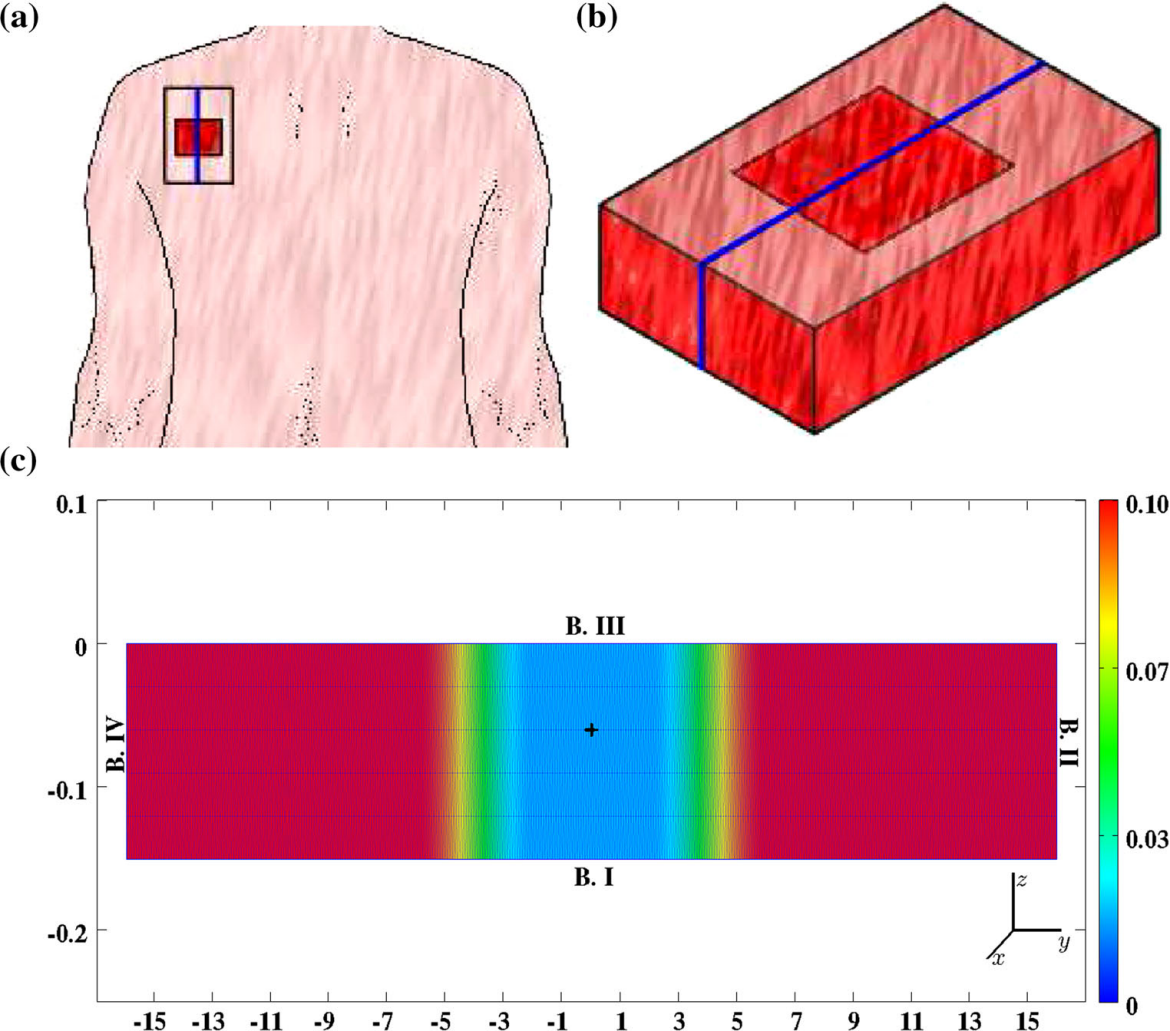
A graphical representation of the domain of computation. **a** A hypothetical wound covering a portion of a shoulder. **b** A close-up of a piece of the dermal layer of the shoulder from **a**. **c** A close-up of the piece of dermal layer from **b** that is enclosed by the *blue box* (the scale along both axes is in centimeters). Depicted are the initial shape of the infinitely thin slice of dermal layer and, in *color scale*, the initial concentration of the collagen molecules, measured in $\text {g}/\text {cm}^{3}$. Within **c**, the boundaries are numbered counterclockwise from *B.I* to *B.IV*. *B.I* coincides with the boundary between the subcutaneous layer and the dermal layer of the skin, *B.II* and *B.IV* border on adjacent dermal tissue, and *B.III* coincides with the boundary between the dermal layer and the epidermal layer (if present). Furthermore, the *black plus sign* located more or less at the center of the wound marks the material point within the dermal layer where the evolution of the individual modeled constituents was traced over time for the generation of the figures in Sect. 5

### The domain of computation

For the generation of simulation results, the computational domain depicted in Fig. 1 has been used. Note that we assume that the exposed surface area of the wound is a perfect rectangle and that the wound extends uniformly to the boundary between the subcutaneous layer and the dermal layer of the skin. The blue box depicted in Fig. 1b coincides with one of the planes of symmetry of the wound. The actual analyses are performed on the slice depicted in Fig. 1c.

The thickness of unwounded dermis is $0.15\ \text {cm}$ in the model. This is in close agreement with the measurements of the thickness of normal skin obtained by Nedelec and colleagues (2014) (Skin tissue actually consists of two layers: an epidermal layer and a dermal layer. Oliveira and colleagues (2005) measured the thickness of the epidermis of normal skin tissue, and their measurements showed that the epidermis of this tissue has an average thickness of less than $95\ \mu \text {m}$. Hence, the thickness of the dermis is more or less equal to the thickness of the epidermis and the dermis combined.).

Using Lagrangian coordinates ($\mathbf {X} = (X,Y,Z)^{\text {T}}$), the domain of computation (${\varOmega }_{X}$) is described mathematically by

20 $$\begin{aligned} {\varOmega }_{X} \in \{X = 0,\ -15.96 \le Y \le 15.96,\ -0.15 \le Z \le 0\}. \end{aligned}$$

Note that $u = 0$, $\partial v /\partial x = 0$ and $\partial w /\partial x = 0$ hold within the domain of computation as a consequence of the present symmetry (with $\mathbf {u} = (u,v,w)^{\text {T}}$). Effectively, this implies that the plane strain assumption holds within the slice of dermal layer (Lai et al. 1999). In addition, the derivatives of the concentrations and the cell densities of the individual constituents, in the *x*-direction, are also zero due to the present symmetry.

### The initial conditions and the boundary conditions

The initial conditions give a description of the various cell densities and the various concentrations at the onset of the proliferative phase of the wound healing cascade. For the generation of simulation results, the following general function has been used to describe the shape of the wound:

21 $$\begin{aligned} w(\mathbf {X})= 1 - \left( 1 - \text {H}_{s}\left( Y,c^{I},c^{II}\right) \right) \text {H}_{s}\left( Y,c^{I},-c^{II}\right) ,\qquad \end{aligned}$$

with

22 $$\begin{aligned} \text {H}_{s}(x,a,b) = {\left\{ \begin{array}{ll} 0 &{} \text {if }\quad x < (b - a), \\ \frac{1}{2}\left( 1 + \sin \left( \frac{(x - b)\pi }{2a}\right) \right) &{} \text {if }\quad |b - x| \le a, \\ 1 &{} \text {if }\quad x > (b + a). \end{array}\right. } \end{aligned}$$

The values of the parameters $c^{I}$ and $c^{II}$ determine, respectively, the steepness of the boundary of the wounded area and the width of the wound. In this study, we take $c^{I} = 2\ \text {cm}$ and $3 \le c^{II} \le 5\ \text {cm}$. Here $w = 0$ corresponds to completely wounded dermis and $w = 1$ corresponds to unwounded dermis. Based on this general function for the shape of the wound, we take the following initial conditions for the modeled constituents of the dermal layer:

23 $$\begin{aligned} \begin{aligned} N(\mathbf {X},0)&= \left( N^{w} + \left( 1 - N^{w}\right) w(\mathbf {X})\right) \overline{N}, \\ M(\mathbf {X},0)&= \overline{M}, \\ c(\mathbf {X},0)&= (1 - w(\mathbf {X}))c^{w}, \\ \rho (\mathbf {X},0)&= \left( \rho ^{w} + \left( 1 - \rho ^{w}\right) w(\mathbf {X})\right) \overline{\rho }, \end{aligned} \end{aligned}$$

where $\overline{N}$, $\overline{M}$ and $\overline{\rho }$ are respectively, the equilibrium cell density of the fibroblast population, the equilibrium cell density of the myofibroblast population, and the equilibrium concentration of the collagen molecules of the unwounded dermis. Due to early secretion of signaling molecules by for instance platelets, signaling molecules are present in the wounded area. The constant $c^{w}$ is the maximum of the initial concentration of the signaling molecule in the wounded area. Furthermore, we assume that there are some fibroblasts and collagen molecules present in the wounded area. The constants $N^{w}$ and $\rho ^{w}$ determine how much fibroblasts and collagen molecules are present minimally in the wounded area.

With respect to the initial conditions for the displacement of the dermal layer, the following holds. The initial cell density of the myofibroblast population is equal to zero everywhere in the domain of computation. Looking at Eq. (9), this implies $\mathbf {f}(\mathbf {x},0) = \mathbf {0}$. Hence

24 $$\begin{aligned} \mathbf {u}(\mathbf {x},0) = \mathbf {0}\quad \forall \mathbf {x} \in {\varOmega }_{x}, \end{aligned}$$

where ${\varOmega }_{x}$ is the domain of computation in Eulerian coordinates, and $\mathbf {x} = (x,y,z)^{\text {T}}$ are the Eulerian coordinates.

With respect to the boundary conditions for the constituents of the dermal layer, we take the following Dirichlet boundary conditions for the second and the fourth boundary

25 $$\begin{aligned} N = \overline{N},\ \ M = \overline{M},\ \ c = \overline{c}, \end{aligned}$$

where $\overline{c}$ is the equilibrium concentration of the signaling molecules in the unwounded dermis. The following Neumann boundary conditions are chosen furthermore for the first and the third boundary

26 $$\begin{aligned} \mathbf {n}\cdot \mathbf {J}_{N} = 0,\ \ \mathbf {n}\cdot \mathbf {J}_{M} = 0,\ \ \mathbf {n}\cdot \mathbf {J}_{c} = 0, \end{aligned}$$

where $\mathbf {n}$ is the unit outward pointing normal vector to the boundary.

With respect to the boundary conditions for the mechanical component of the model, we take the following Robin boundary conditions

27 $$ \begin{aligned}&\text {B.I:}\ \ \mathbf {n}\cdot \mathbf {\sigma } = \begin{bmatrix} 0 \\ 0 \\ -s_{1}\rho w \end{bmatrix},\ \ \text {B.III:}\ \ \mathbf {n}\cdot \mathbf {\sigma } = \begin{bmatrix} 0 \\ 0 \\ 0 \end{bmatrix}, \nonumber \\&\quad \text {B.II } \& \text {B.IV:}\ \ \mathbf {n}\cdot \mathbf {\sigma } = \begin{bmatrix} 0 \\ -s_{2}\rho v \\ 0 \end{bmatrix}. \end{aligned}$$

These boundary conditions imply that the first boundary is free to move in the direction of the *x*-axis and the *y*-axis, while it experiences an opposing spring-like force per unit area in the direction of the *z*-axis that is proportional to the concentration of the collagen molecules and the displacement in the direction of the *z*-axis. With respect to the second and fourth boundary, the boundary conditions imply that these boundaries are free to move in the direction of the *x*-axis and the *z*-axis, while they experience an opposing spring-like force per unit area in the direction of the *y*-axis that is proportional to the concentration of the collagen molecules and the displacement in the direction of the *y*-axis. The boundary condition for the third boundary implies that this boundary is free to move in any direction.

### The parameter value estimates

Most of the parameter values were estimated on the basis of previously conducted studies. Furthermore, we could determine some parameter values due to the fact that these values are a necessary consequence of the values chosen for other parameters. We elaborate on this in Appendix 1. The few remaining values were based on educated guesses and preliminary numerical simulations. See Table 1 in Appendix 1 for an overview of the dimensional values of the parameters.

## Numerical algorithm

For the kernel of the concrete expression of the algorithm, we used MATLAB together with MATLAB’s Parallel Computing Toolbox (The MathWorks Inc. 2014). Furthermore, we used a slightly adapted version of the mesh generator developed by Persson and Strang (2004) and the scaling and permutation routine HSL_MC64 (HSL 2013) by interfacing these products with the kernel. Finally, we applied the non-dimensionalization presented in Appendix 2, to the model.

The algorithm consists of two parts. The first part of the algorithm is dedicated to the generation of a conforming triangulation of the domain of computation. For this end, we used a slightly adapted version of the aforementioned mesh generator. This resulted in high-quality meshes that consist mainly of equilateral triangles. The only triangles that were not equilateral were located near the second and the fourth boundary of the domain of computation. These latter triangles were nearly equilateral. Using the following measure for the quality of a triangle *ABC*:

28 $$\begin{aligned} \alpha (ABC) = 2\sqrt{3}\left( \frac{\Vert CA \times CB\Vert }{\Vert CA\Vert ^{2} + \Vert AB\Vert ^{2} + \Vert BC\Vert ^{2}}\right) , \end{aligned}$$

we observed that $\alpha > 0.85$ for all triangles in the triangulation, where $0 \le \alpha \le 1$ and $\alpha = 1$ for equilateral triangles (Lo 1989). For the generation of the simulation results presented in Sect. 5, we used a triangulation consisting of triangles with an average initial edge length of $3.46 \times 10^{-2}\ \text {cm}$. We repeated the calculations two times: the first time we used a triangulation consisting of triangles with an average initial edge length of $5.77 \times 10^{-2}\ \text {cm}$, and the second time we used a triangulation consisting of triangles with an average initial edge length of $1.73 \times 10^{-2}\ \text {cm}$. We observed that the difference in the simulation results with respect to the outcomes between the different calculations was negligible.

The second part of the algorithm is dedicated to obtaining an approximation of the solution for the displacement and the modeled constituents of the dermal layer from system (1), after application of the non-dimensionalization. In order to solve the time-dependent problem, the method of lines and the standard fixed-point defect correction method were used (Van Kan et al. 2014). The two equations of the system are solved in a segregated way. At each time step, an approximation of the solution for the modeled constituents of the dermal layer is determined first, and subsequently, an approximation of the solution for the displacement of the dermal layer is determined. This scheme is iterated until certain convergence criteria are met (The relative residuals of the approximations must be below a certain predefined upper bound and the relative difference between subsequent approximations must be below a certain predefined upper bound.). The required estimate of the gradient of the solution for the signaling molecule is obtained by using a variational gradient recovery projection scheme (Lyra 1994).

**Fig. 2:**
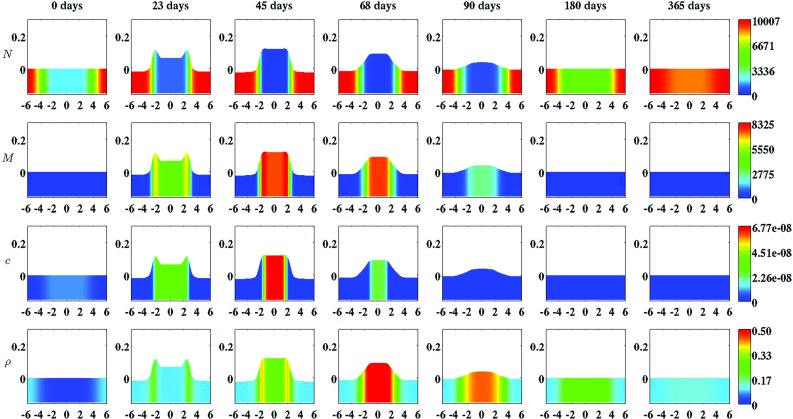
An overview of a simulation with a relatively high apoptosis rate of myofibroblasts ($\delta _{M} = 6\times 10^{-2}\ /\text {day}$). The *first two rows* show the evolution over time of the cell density of, respectively, the fibroblast population and the myofibroblast population. The *color scales* represent the cell densities, measured in $\text {cells}/\text {cm}^{3}$. The *last two rows* show the evolution over time of the concentration of, respectively, the signaling molecules and the collagen molecules. The *color scales* represent the concentrations, measured in $\text {g}/\text {cm}^{3}$. Within the subfigures, the scale along both axes is in centimeters. All remaining parameter values are equal to those depicted in Table 1 in Appendix 1. With respect to the width of the wound, $c^{II} = 4\, \text {cm}$ in this simulation

**Fig. 3:**
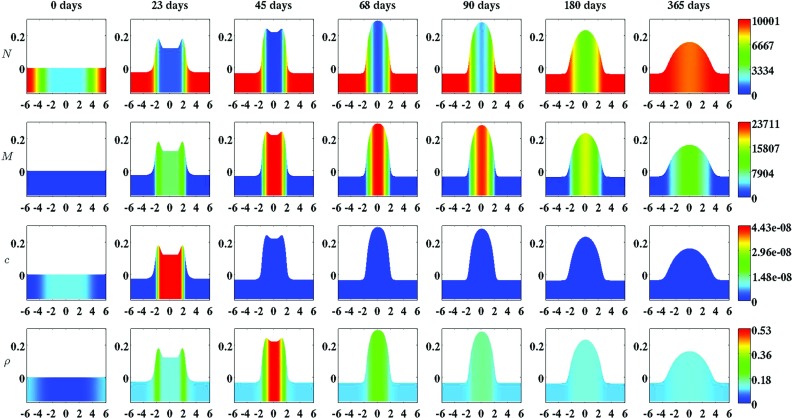
An overview of a simulation with a relatively low apoptosis rate of myofibroblasts ($\delta _{M} = 2\times 10^{-3}\ /\text {day}$). The *first two rows* show the evolution over time of the cell density of, respectively, the fibroblast population and the myofibroblast population. The *color scales* represent the cell densities, measured in $\text {cells}/\text {cm}^{3}$. The *last two rows* show the evolution over time of the concentration of, respectively, the signaling molecules and the collagen molecules. The *color scales* represent the concentrations, measured in $\text {g}/\text {cm}^{3}$. Within the subfigures, the scale along both axes is in centimeters. All remaining parameter values are equal to those depicted in Table 1 in Appendix 1. With respect to the width of the wound, $c^{II} = 4\,\text {cm}$ in this simulation

For the discretization of the system of equations, a moving-grid finite-element method was used (Madzvamuse et al. 2003) together with the first-order backward Euler time-integration method. Furthermore, a semi-implicit flux-corrected transport (FCT) limiter developed by Möller and colleagues (2008) and a source term splitting procedure proposed by Patankar (1980) are applied on the discretized system of equations that describe the dynamics of the modeled constituents of the dermal layer. Taken together, these latter two techniques enforce positivity of the approximations of the solutions for the constituents of the dermal layer.

The individual time steps are chosen by using an automatically adaptive time-stepping method with inbuilt local truncation error control (Kavetski et al. 2002). After obtaining and accepting an approximation for a certain time step, the local extrapolation procedure proposed by Kavetski and colleagues is applied to increase the accuracy of this approximation.

For the approximation of the individual primary variables of the model, functions from the space of triangular finite elements with linear basis functions were chosen (Quarteroni and Valli 2008). The integrals over the interior of the elements are approximated by a second-order accurate Newton–Cotes quadrature rule, and the integrals over the border of the elements are approximated by a second-order accurate Gaussian quadrature rule.

In order to obtain approximate solutions for the resulting linear systems of equations, MATLAB’s backslash operator is used (The MathWorks Inc. 2014) after using MATLAB’s LU factorization algorithm (Davis and Duff 1997) on scaled and permuted versions of the original linear systems. For the scaling and permutation of the linear systems, several inbuilt scaling and permutation algorithms of MATLAB are used (Davis et al. 2004; Duff and Koster 1999) together with the scaling and permutation routine HSL_MC64 (HSL 2013).

## Simulation results

**Fig. 4:**
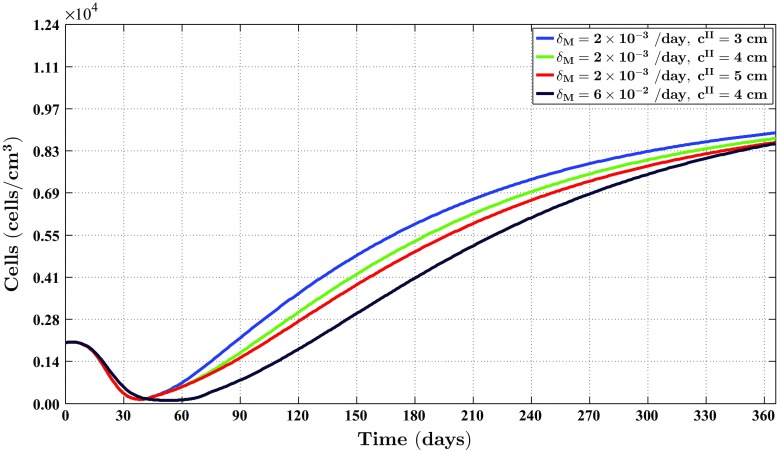
The evolution over time of the cell density of the fibroblast population for different values of the apoptosis rate of myofibroblasts and various widths of the wound. See Fig. 1c for the location where the evolution of the cell density was traced over time. The remaining parameter values are equal to those depicted in Table 1 in Appendix 1

**Fig. 5:**
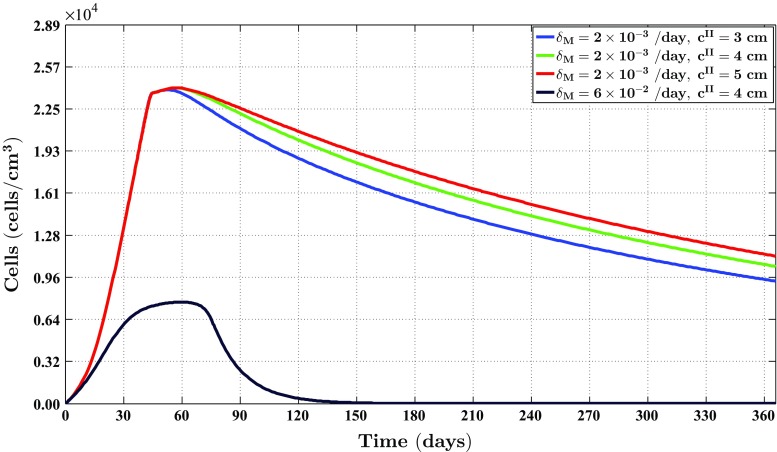
The evolution over time of the cell density of the myofibroblast population for different values for the apoptosis rate of myofibroblasts and various widths of the wound. See Fig. 1c for the location where the evolution of the cell density was traced over time. The remaining parameter values are equal to those depicted in Table 1 in Appendix 1

**Fig. 6:**
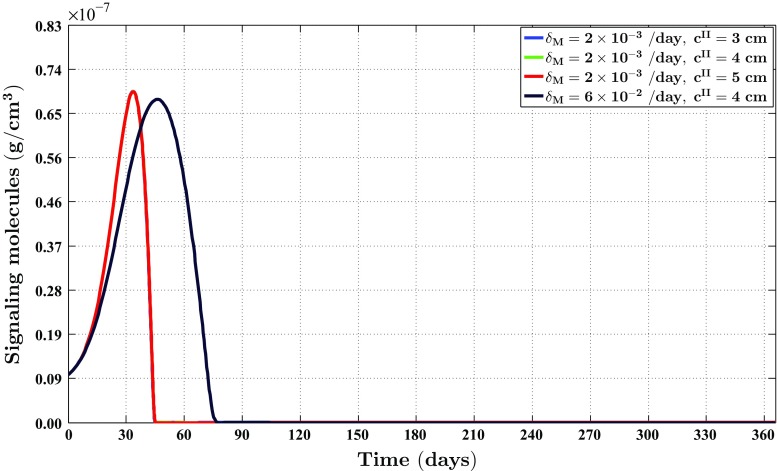
The evolution over time of the concentration of the signaling molecules for different values for the apoptosis rate of myofibroblasts and various widths of the wound (the *blue curve* and the *green curve* are situated underneath the red curve). See Fig. 1c for the location where the evolution of the concentration was traced over time. The remaining parameter values are equal to those depicted in Table 1 in Appendix 1

**Fig. 7:**
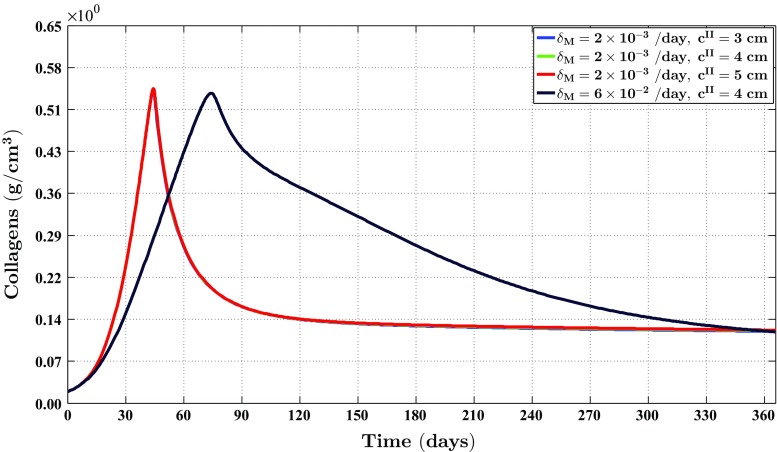
The evolution over time of the concentration of the collagen molecules for different values for the apoptosis rate of myofibroblasts and various widths of the wound (the *blue curve* and the *green curve* are situated underneath the *red curve*). See Fig. 1c for the location where the evolution of the concentration was traced over time. The remaining parameter values are equal to those depicted in Table 1 in Appendix 1

**Fig. 8:**
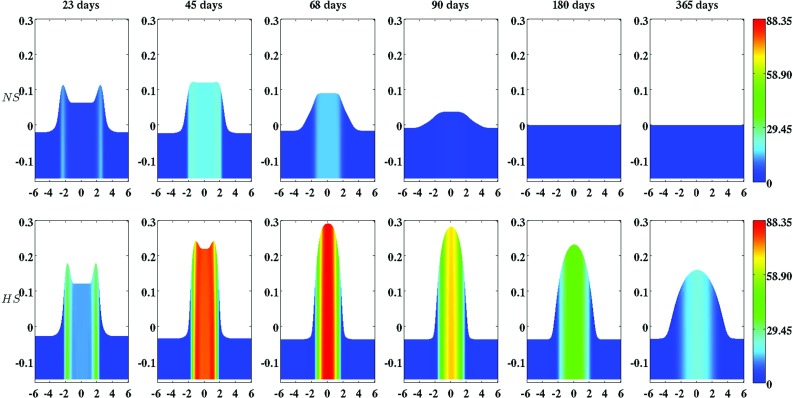
The evolution over time of the strain energy density. The *first row* shows the evolution when the apoptosis rate of myofibroblasts is relatively high ($\delta _{M} = 6\times 10^{-2}\ /\text {day}$). The *second row* shows the evolution when the apoptosis rate of myofibroblasts is relatively low ($\delta _{M} = 2\times 10^{-3}\ /\text {day}$). With respect to the width of the wound, $c^{II} = 4\ \text {cm}$ in both cases. The *color scales* represent the strain energy density, measured in $\text {J}/\text {cm}^{3}$. Within the subfigures, the scale along both axes is in centimeters. The remaining parameter values are equal to those depicted in Table 1 in Appendix 1

**Fig. 9:**
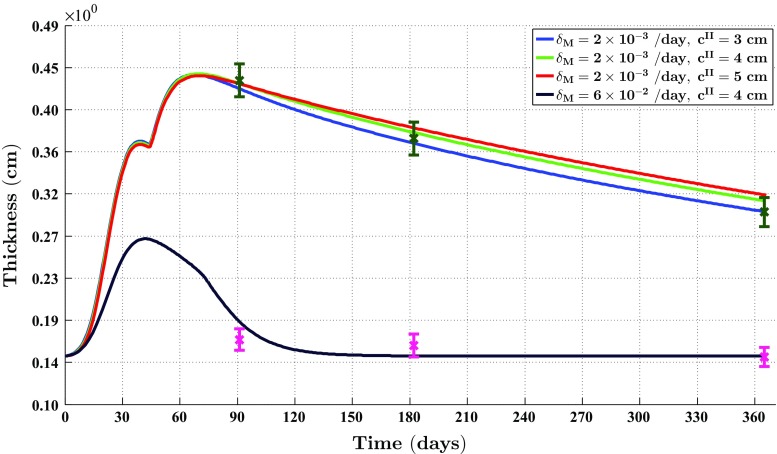
The evolution over time of the thickness of the dermal layer for different values for the apoptosis rate of myofibroblasts and various widths of the wound. In the simulations, the thickness of the dermal layer was computed at $y = 0\, \text {cm}$. The *dark green error bars* and the *magenta error bars* represent clinical measurements of the thickness of, respectively, hypertrophic scars and normal scars in human subjects at different time points after injury (Nedelec et al. 2014). Displayed are the means (with a *cross sign*) *plus*/*minus* one standard deviation. The remaining parameter values are equal to those depicted in Table 1 in Appendix 1

Several studies have demonstrated that the cell density of myofibroblasts in hypertrophic scar tissue is raised compared to the cell density of myofibroblasts in normal scar tissue (Van der Veer et al. 2009). Furthermore, Desmoulière and colleagues (1995) have suggested that the disruption of apoptosis during wound healing might be an important factor in the development of pathological scarring. Based on this and given that the apoptosis rate of myofibroblasts is a free parameter of the model, we decided to modify the value of the apoptosis rate of myofibroblasts over simulations in order to investigate the influence of such modifications on the healing process. Furthermore, we were also interested in what the effect is of varying the width of the wound [i.e., varying the value of the parameter $c^{II}$ (See Sect. 3.7)]. Therefore, we also varied the width of the wound over simulations. Figure 2 shows an overview of a simulation with a relatively high apoptosis rate and $c^{II} = 4\ \text {cm}$. Figure 3 shows an overview of a simulation with a relatively low apoptosis rate and $c^{II} = 4\ \text {cm}$.

Figures 4, 5, 6 and 7 show the evolution over time of the different cell densities and the different concentrations for either a relatively high apoptosis rate of myofibroblasts or a relatively low apoptosis rate of myofibroblasts, and various widths of the wound, at a certain material point within the wounded area. In order not to clutter the figures too much, we did not include the simulation data into the figures where the apoptosis rate is relatively high, and the width of the wound is either relatively small (i.e., $c^{II} = 3\ \text {cm}$) or relatively large (i.e., $c^{II} = 5\ \text {cm}$). These data are actually very similar to the presented data where the apoptosis rate of the myofibroblasts is relatively high and $c^{II} = 4\ \text {cm}$.

Figure 8 shows the evolution over time of the strain energy density for both a relatively high apoptosis rate of myofibroblasts and a relatively low apoptosis rate of myofibroblasts. The strain energy density (*W*) was determined by computing

29 $$\begin{aligned} W = C_{1}\left( \overline{I}_{1} - 3\right) + D_{1}\left( J - 1\right) ^{2}, \end{aligned}$$

at various time points ($\overline{I}_{1} = J^{-\frac{2}{3}}\text {tr}\left( \mathbf {B}\right)$).

Figure 9 shows the evolution over time of the thickness of the dermal layer for different values for the apoptosis rate of myofibroblasts and various widths of the wound. Once more, in order not to clutter the figure too much, we did not include the simulation data into the figure where the apoptosis rate is relatively high, and the width of the wound is either relatively small or relatively large. These data are actually very similar to the presented data where the apoptosis rate of myofibroblasts is relatively high and $c^{II} = 4\ \text {cm}$. The error bars represent clinical measurements of the thickness of hypertrophic scars and normal scars in human subjects at different time points after injury (Nedelec et al. 2014).

Figures 2, 3, 4, 5, 6, 7, 8 and 9 show clearly that the value for the apoptosis rate of myofibroblasts has a huge impact on the healing response. That is, if the apoptosis rate is relatively high, then simulations show gentle healing; the maximum myofibroblasts cell density in the wounded area is relatively low, and the restoration of the presence of a collagen-rich ECM in the wounded area is more gradual. The degree of compaction (i.e., the degree of wound contraction) remains relatively low, and the dermal layer remains relatively thin.

If the apoptosis rate is relatively low, then simulations show an excessive healing response; the maximum myofibroblasts cell density in the wounded area is relatively high, and the restoration of the presence of a collagen-rich ECM in the wounded area is accomplished faster. During the remodeling of the evolving ECM, the cell density of the myofibroblast population diminishes slowly, while the cell density of the fibroblast population increases slowly toward the equilibrium cell density. The degree of temporary compaction is relatively high during the execution of the wound healing processes compared to the situation where the apoptosis rate is relatively high, as is demonstrated in Fig. 8 by the relatively high strain energy density in the wounded area. Furthermore, the restoring dermal tissue becomes quite a bit thicker than the unaffected surrounding tissue. Over time, the thickness of the dermal layer declines slowly toward the thickness of unwounded dermis. Finally, the figures also show that the width of the wounded area has a relatively small, but clear effect on the evolution of the cell densities within the wounded area and on the evolution of the thickness of the dermal layer in the case of a relatively low apoptosis rate.

Figure 9 also shows a good match with respect to the evolution of the thickness of the dermal layer between the outcomes of different simulations with different values for the apoptosis rate of myofibroblasts and clinical measurements of the thickness of both hypertrophic and normal scars at different time points after injury in humans. Oliveira and colleagues (2005) measured the thickness of the epidermises of both normal and hypertrophic scar tissue, and their measurements showed that the epidermises of these tissues have an average thickness of, respectively, less than $121\ \mu \text {m}$ and $166\ \mu \text {m}$. Hence, the thickness of the dermises of these tissues is more or less equal to the thickness of their epidermis and dermis combined.

## Discussion

We have presented a new model for the simulation of the formation and the subsequent regression of hypertrophic scar tissue after dermal wounding. We consider the following components as the core components of the model. The first component is the interval of time of the wound healing response that is modeled; basically, this interval of time encloses the proliferative phase and a large portion of the remodeling phase. We consider the modeling of the dermal layer as a heterogeneous, isotropic and compressible neo-Hookean solid as the second core component of the model. The third component is the mechanical force balance between the generated stresses by the myofibroblast population and the intrinsic restoring stresses of the dermal layer. The fourth component is the active infiltration of fibroblasts and myofibroblasts into the wounded area under the influence of signaling molecules. The fifth component is the cell differentiation of fibroblasts into myofibroblasts. The sixth component is the secretion and the degradation of both signaling molecules and collagen molecules. Within the model, the degradation of these molecules is modeled as proteolytic cleavage by a generic MMP. The seventh component is the flexible and realistic shape of the modeled dermal layer. The final core component is the novel description of the mechanical interactions between the modeled piece of dermal layer and its adjacent structures.

Furthermore, we have presented a broad overview of the custom-made numerical algorithm that has been developed for the generation of computer simulations with the model. The development of this algorithm was necessary in order to guarantee the positivity of the approximations of the solutions for the constituents of the dermal layer and obtain sufficiently accurate simulations sufficiently fast.

With the presented model, it is possible in general to simulate some qualitative features of the dermal wound healing response. The temporary presence of myofibroblasts in the wounded area during the execution of the wound healing response can be simulated. Furthermore, it is possible to simulate the contraction and the subsequent retraction of the wounded area, and it is possible to simulate the restoration of the presence of fibroblasts and a collagen-rich ECM in the recovering wounded area.

With respect to the simulation of the formation and the subsequent regression of hypertrophic scar tissue, we observe the following. The value for the apoptosis rate of myofibroblasts has a huge impact on the healing response. Figure 5 shows that there exists a strong connection between the size of the value for the apoptosis rate of myofibroblasts and the maximum cell density of the myofibroblast population in the wounded area. Furthermore, Fig. 7 shows that the size of the value for the apoptosis rate of myofibroblasts also strongly influences the dynamics related to the collagen molecules. Given Eq. (9), this implies that the size of the value for the apoptosis rate of myofibroblasts has a huge impact on the total generated stress by the myofibroblast population and hence the stored strain energy in the wounded area. Consequently, there exists a strong connection between the size of the value for the apoptosis rate of myofibroblasts and the thickness of the wounded area, as is confirmed by the results depicted in Fig. 9. Hence, provided that the size of the value for the apoptosis rate of myofibroblasts is small enough, it is indeed possible with the model to simulate some features of the formation and the subsequent regression of hypertrophic scar tissue. In addition, the width of the simulated wound has a relatively small, but clear effect on the evolution of the cell densities within the wounded area and on the evolution of the thickness of the dermal layer.

Figure 9 also displays a good match with respect to the evolution of the thickness of the dermal layer between the outcomes of different simulations with different values for the apoptosis rate of myofibroblasts and clinical measurements of the thickness of both hypertrophic and normal scars at different time points after injury in humans (Nedelec et al. 2014). Interestingly, the figure shows that a relatively high apoptosis rate of myofibroblasts results in scar tissue that behaves like normal scar tissue with respect to the evolution of the thickness of the tissue over time, while a relatively low apoptosis rate results in scar tissue that behaves like hypertrophic scar tissue with respect to the evolution of the thickness of the tissue over time. This is in agreement with the suggestion put forward by Desmoulière and colleagues (1995) that the disruption of apoptosis during wound healing might be an important factor in the development of pathological scarring.

Irrespective of the value for the apoptosis rate of myofibroblasts, the thickness of the scar tissue will start to decline gradually as the cell density of the myofibroblast population diminishes slowly, and ultimately, the properties of the scar tissue will be identical to the properties of the surrounding tissue. This makes perfect sense from a mathematical point of view and is more or less in agreement with clinical observations (Hawkins and Finnerty 2012). A simple mathematical analysis demonstrates that the only stable equilibrium solutions for the cell densities and the concentrations of the individual constituents of the dermal layer are constant over the domain of computation (i.e., the equilibrium solutions are not dependent on the spatial variable). This implies that the “body force” in the mechanical force balance vanishes when the solutions related to the constituents of the dermal layer reach their equilibrium solutions. Subsequently, this implies that the displacement field of the dermal layer becomes zero when the solutions related to the constituents of the dermal layer reach their equilibrium solutions. Consequently, the properties of the scar tissue will indeed become identical to the properties of its surrounding tissue. However, note that the cell density of the myofibroblast population declines very slowly toward the equilibrium concentration when the value of the apoptosis rate is relatively low (see Fig. 5). Given Eq. (9), this implies that the dermal layer remains relatively thick for a prolonged period of time.

As has been mentioned in the introduction, our ultimate goal is to construct models with which the properties of newly generated tissues that form during the healing of dermal wounds, can be predicted with a high degree of certainty. With the presented model, it is possible to simulate some properties of hypertrophic scar tissue such as the relatively high cell density of the myofibroblast population and the relatively large thickness of the dermal layer. However, other characteristic properties of hypertrophic scar tissue like the often observed (dark) reddish, hyperpigmented appearance cannot be simulated with the presented model. Sometimes, the formation of hypertrophic scar tissue also coincides with the formation of contractures (i.e., permanent contractions) (Hawkins and Finnerty 2012). Given that ultimately the properties of the recovering injured area will be identical to the properties of the surrounding tissue, it is not possible to simulate the concurrent formation of such contractures. Hence, the road toward the construction of widely employable predictive models is still long and daunting, but we consider the development of the presented model and the accompanying numerical algorithm as further steps toward their construction.

**Table 1:** An overview of the dimensional values of the parameters of the model together with a reference to the source of the used data set for their estimation, if available

Parameter	Value	Dimensions	Reference
$E^{I}$	$$3.2\times 10\ $$	$(\text {N})/((\text {g cm})^{1/2} )$	Liang and Boppart (2010)
$\nu$	$$4.9\times 10^{-1}\ $$	−	Liang and Boppart (2010)
$\xi$	$$2\times 10^{-3}\ $$	$(\text {N g})/(\text {cells cm}^{2})$	Maskarinec et al. (2009) and Wrobel et al. (2002)
*R*	$$3\times 10^{-1}\ $$	$\text {g}/\text {cm}^{3}$	Olsen et al. (1995b)
$D_{F}$	$$10^{-7}\ $$	$\text {cm}^{5}/\text {cells day}$	Sillman et al. (2003)
$\chi _{F}$	$$2\times 10^{-3}\ $$	$\text {cm}^{5}/\text {g day}$	Murphy et al. (2012)
*p*	$$-4.2\times 10^{-1}\ $$	−	NC
$r_{F}$	$$9.24\times 10^{-1}\ $$	$\text {cm}^{3p}/(\text {cells}^{p}\ \text {day})$	Ghosh et al. (2007)
$r_{F}^{\max }$	$$2\ $$	−	Strutz et al. (2001)
$a_{c}^{I}$	$$10^{-8}\ $$	$\text {g}/\text {cm}^{3}$	Grotendorst (1992)
$\kappa _{F}$	$$10^{-6}\ $$	$\text {cm}^{3}/(\text {cells})$	Vande Berg et al. (1989)
$k_{F}$	$$5.4\times 10^{6}\ $$	$\text {cm}^{3}/(\text {g day})$	Desmoulière et al. (1993)
$\delta _{N}$	$$2\times 10^{-2}\ $$	$/\text {day}$	Olsen et al. (1995b)
$\delta _{M}$	$$(2 - 60)\times 10^{-3}\ $$	$/\text {day}$	TW
$D_{c}$	$$2.9\times 10^{-3}\ $$	$\text {cm}^{2}/\text {day}$	Murphy et al. (2012)
$k_{c}$	$$4\times 10^{-13}\ $$	$\text {g}/(\text {cells day})$	Olsen et al. (1995b)
$\eta$	$$2\ $$	−	Rudolph and Vande Berg (1991) and Moulin et al. (1998)
$a_{c}^{II}$	$$10^{-8}\ $$	$\text {g}/\text {cm}^{3}$	Olsen et al. (1995b)
$\delta _{c}$	$$5\times 10^{-4}\ $$	$\text {cm}^{6}/(\text {cells g day})$	Olsen et al. (1995b)
$a_{c}^{III}$	$$2\times 10^{8}\ $$	$\text {cm}^{3}/\text {g}$	Overall et al. (1991)
$k_{\rho }$	$$6\times 10^{-8}\ $$	$\text {g}/(\text {cells day})$	NC
$k_{\rho }^{\max }$	$$10\ $$	−	Olsen et al. (1995b)
$a_{c}^{IV}$	$$10^{-9}\ $$	$\text {g}/\text {cm}^{3}$	Roberts et al. (1986)
$\delta _{\rho }$	$$6\times 10^{-6}\ $$	$\text {cm}^{6}/(\text {cells g day})$	TW
$\overline{N}$	$$10^{4}\ $$	$\text {cells}/\text {cm}^{3}$	Olsen et al. (1995b)
$\overline{M}$	$$0\ $$	$\text {cells}/\text {cm}^{3}$	Olsen et al. (1995b)
$\overline{c}$	$$0\ $$	$\text {g}/\text {cm}^{3}$	NC
$\overline{\rho }$	$$10^{-1}\ $$	$\text {g}/\text {cm}^{3}$	Olsen et al. (1995b)
$N^{w}$	$$2\times 10^{-1}\ $$	−	TW
$\rho ^{w}$	$$2\times 10^{-1}\ $$	−	TW
$c^{w}$	$$10^{-8}\ $$	$\text {g}/\text {cm}^{3}$	Olsen et al. (1995b)
$s_{1}$	$$10^{6}\ $$	$\text {N}/\text {g}$	Javierre et al. (2009)
$s_{2}$	$$10^{3}\ $$	$\text {N}/\text {g}$	TW

If a parameter value has been estimated in this study, but not on the basis of data from previous studies, then this is indicated by the abbreviation TW. If a parameter value is a consequence of the values chosen for the other parameters, then this is indicated by the abbreviation NC

## Appendix 1: The parameter value estimates

Three parameter values are a consequence of the values chosen for the other parameters. These are the equilibrium signaling molecule concentration of the unwounded dermis ($\overline{c}$), the constant *p* and the collagen molecule secretion rate ($k_{\rho }$). If we take $\overline{\rho } = 0.1 \ \text {g}/\text {cm}^{3}$, $\overline{N} = 10^{4}\ \text {cells}/\text {cm}^{3}$ and $\overline{M} = 0\ \text {cells}/\text {cm}^{3}$ in the unwounded dermis, then $c = 0\ \text {g}/\text {cm}^{3}$ is an attracting equilibrium in the vicinity of $c = 0\ \text {g}/\text {cm}^{3}$. Hence, we take $\overline{c} = 0\ \text {g}/\text {cm}^{3}$. Furthermore, if $\overline{N} = 10^{4}\ \text {cells}/\text {cm}^{3}$, $\overline{M} = 0\ \text {cells}/\text {cm}^{3}$ and $\overline{c} = 0\ \text {g}/\text {cm}^{3}$, then $p = (\log (\delta _{N}) - \log (r_{F}(1 - \kappa _{F}\overline{N})))/\log (\overline{N})$. Finally, if $\overline{M} = 0\ \text {cells}/\text {cm}^{3}$ and $\overline{c} = 0\ \text {g}/\text {cm}^{3}$, then $k_{\rho } = \delta _{\rho }\overline{\rho }^2\ \text {g}/(\text {cells}\ \text { day})$. See Table 1 for an overview of the dimensional values of all parameters.

## Appendix 2: The non-dimensionalization of the model

We applied the following non-dimensionalization to the model:

$$\begin{aligned} \begin{aligned} x&= Lx^{*},&t&= \left( L^{2}/\left( D_{N}\overline{N}\right) \right) t^{*},&\rho&= \overline{\rho }\rho ^{*}, \\ N&= \overline{N}N^{*},&M&= \overline{N}M^{*},&c&= c^{w}c^{*}, \\ \mathbf {u}&= L\mathbf {u}^{*},&\mathbf {v}&= \left( \left( D_{N}\overline{N}\right) /L\right) \mathbf {v}^{*},&\mathbf {\sigma }&= \left( \left( \xi \overline{N}\right) /\overline{\rho }\right) \mathbf {\sigma }^{*}, \end{aligned} \end{aligned}$$

where $L = 1\ \text {cm}$ is the length scale of the model. The variables with the asterisks are the non-dimensionalized variables.
